# The Road Less Taken: Less Appreciated Pathways for Manipulating CD8^+^ T Cell Exhaustion

**DOI:** 10.3389/fimmu.2022.926714

**Published:** 2022-07-06

**Authors:** Andrea C. Pichler, Jennifer L. Cannons, Pamela L. Schwartzberg

**Affiliations:** ^1^ Laboratory of Immune System Biology, National Institute of Allergy and Infectious Diseases, National Institutes of Health, Bethesda, MD, United States; ^2^ National Human Genome Research Institute, National Institutes of Health, Bethesda, MD, United States

**Keywords:** CD8^+^ T cell exhaustion, CD226, CD137, TCF-1, PI3 Kinase delta, IL-2, metabolism

## Abstract

Exhausted CD8^+^ T (Tex) cells are a distinct cell population that arise during persistent antigen exposure in the context of chronic infections and cancers. Although characterized by progressive loss of effector functions, high and sustained inhibitory receptor expression and distinct transcriptional and epigenetic programs, Tex cells are heterogeneous. Among these, a self-renewing TCF-1^+^ Tex population, having unique characteristics and the ability to respond to immune-checkpoint blockade, gives rise to TCF-1^-^ terminally Tex cells. These TCF-1^+^ cells have stem cell-like properties similar to memory T cell populations, but the signals that regulate the developmental pathways and relationships among exhausted cell populations are still unclear. Here, we review our current understanding of Tex cell biology, and discuss some less appreciated molecules and pathways affecting T cell exhaustion. We highlight two co-stimulatory receptors, CD226 and CD137, and their role in inducing or restraining T cell exhaustion, as well as signaling pathways that may be amenable to pharmacological inhibition with a focus on Phosphoinositide-3 Kinase and IL-2 partial agonists. Finally, we discuss novel methods that may increase TCF-1^+^ populations and therefore improve immunotherapy responsiveness. Understanding features of and pathways to exhaustion has important implications for the success of immunotherapy, including checkpoint blockade and adoptive T-cell transfer therapies.

## Introduction

CD8^+^ T cells play critical roles in both fighting infection and restraining tumor growth. Activation of CD8^+^ T cells occurs upon the engagement of the T cell receptor (TCR) complex that recognizes foreign or tumor antigens presented by MHC Class I molecules, in conjunction with co-receptors that enhance or diminish TCR signaling.

During acute infection, CD8^+^ T cells can adopt several fates: they can become cytolytic short-lived or long-lived effector cells that help clear infections; alternatively, they can differentiate into memory-precursor cells that form long-lived central and effector memory cells poised for future protection (1). In contrast, during chronic infections and cancer, chronically stimulated antigen-specific T cells progressively decrease in quantity and function as they enter a state of hyporesponsivness called “T cell exhaustion”, characterized by the loss of cytokine production and proliferative potential, development of metabolic dysfunction and increased expression of inhibitory receptors (IRs), including PD-1, Tim-3 and CTLA-4. Targeting these IRs has been validated as a promising therapeutic strategy against cancer, and potentially chronic infection, as illustrated by clinical success achieved with immune checkpoint blockade (ICB) using monoclonal antibodies (mAbs) against PD-1 and CTLA-4 in metastatic melanoma (2, 3). Although exhausted T (Tex) cells display impaired responses to TCR engagement, this hyporesponsive state enables Tex cells to persist under conditions of chronic stimulation (4). Extensive efforts have focused on understanding cellular and molecular mechanisms that drive T cell exhaustion, and finding potential strategies to recover and maintain effector T cell function under conditions of exhaustion.

**Figure 1:**
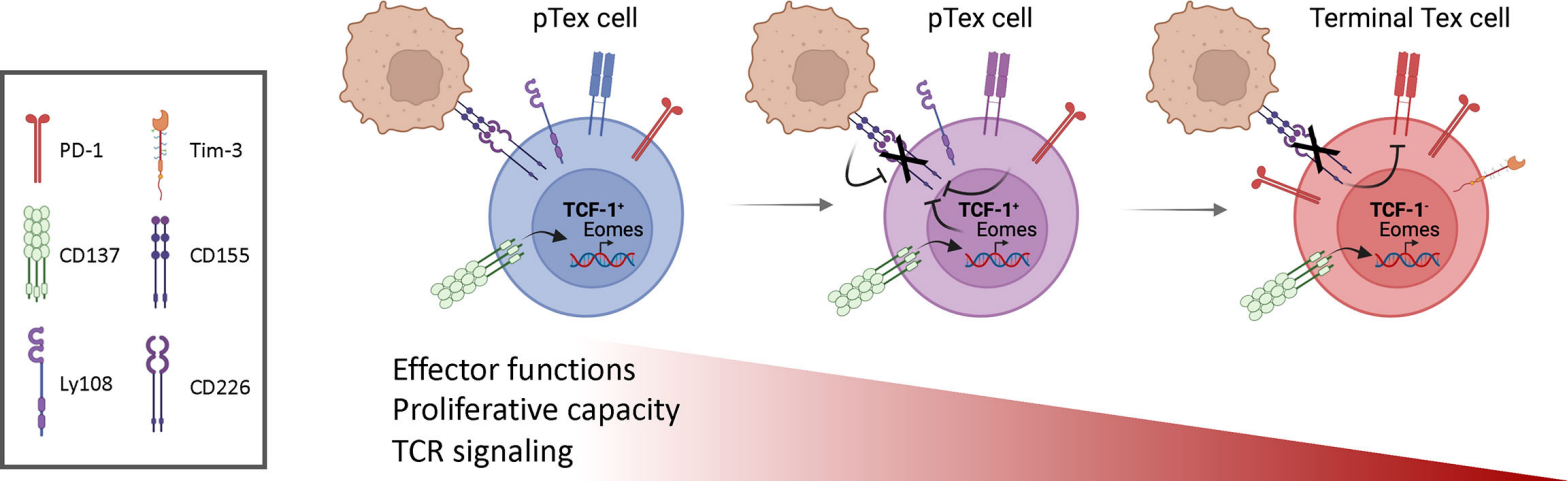
Progressive changes in T cell exhaustion. Stem-like or progenitor exhausted cells (pTex) self-renew and, in the presence of continual antigen stimulation, give rise to more exhausted T (Tex) cells, characterized by expression of inhibitory receptors, loss of effector cell functions, proliferative capacity and their ability to respond to ICB. CD137 stimulation of early pTex cells induces high expression of Eomes, which, together with posttranslational mechanisms induced by CD155 expressed on cancer cells, drives CD226 downregulation. Loss of CD226 further impairs TCR signaling and contributes to T cell dysfunction. Figures drawn using Biorender.

Nonetheless, heterogeneity has been observed among Tex cells, related to the progressive nature of this process. A specific subset of Tex cells, defined as ‘precursor exhausted’ or ‘stem-like’ progenitor (pTex) cells, retains some effector function and shares characteristics with memory cells (**Figure 1**). pTex cells are defined by and require the expression of the transcription factor TCF-1, which is critical for T cell ‘stemness’ (5–7) and is essential for the development of central memory T cells during acute infection (7, 8). TCF-1^+^ pTex cells both self-renew and, upon persistent antigen stimulation, convert into more ‘terminally exhausted’ states, as well as cytolytic effector-like cells (9). Thus, pTex cells are critical to maintain CD8^+^ T cells under conditions of exhaustion. Data argue that pTex cells, as opposed to the bulk of Tex cells that do not express TCF-1, play an indispensible role in immunotherapy, since this population is required for and correlates with efficient responses to ICB (5–7, 10, 11).

Recent studies have revealed that Tex cells also acquire a distinct epigenetic state. The transcription factor Tox is central to this process *via* its role in epigenetic remodeling and transcriptional cascades that orchestrate Tex cell development by directing histone acetylation (12–14). These conserved epigenetic features in terminal exhaustion become fixed and can persist independently of chronic antigen stimulation and inflammation (15–17): this exhausted state cannot be rescued. In contrast, the transcriptional repressor BACH2 is transcriptionally and epigenetically active in TCF-1^+^ pTex cells and has been shown to suppress the molecular program driving terminal exhaustion (18, 19). Since only a fraction of cancer patients’ respond to current ICB such as anti-PD-1 mAbs, therapeutic efforts to recover Tex effector functions may require new approaches including those that increase epigenetic plasticity of Tex cells and promote pTex cells.

In this review, we focus on some less-appreciated pathways affecting the development of T cell exhaustion. We discuss two co-stimulatory receptors, CD226 and CD137, as well as signaling pathways that may be amenable to pharmacological inhibition with a focus on Phosphoinositide-3 Kinase (PI3K) and IL-2 partial agonists and their complex roles in T cell function and exhaustion. Finally, we discuss novel methods that may promote TCF-1^+^ populations and potentially enhance immunotherapy responsiveness. Understanding molecular pathways that contribute to exhaustion has important implications for improving successful immunotherapy, including both ICB and adoptive T cell transfer approaches.

## The Role of Activating Receptors in T Cell Exhaustion

The original two signal model for lymphocyte activation states that T cells require both antigen-receptor engagement and co-stimulatory signals to achieve appropriate activation following interaction with activated antigen presenting cells. Since this original hypothesis and early experiments supporting this concept were published, several decades of work have elucidated an important diversity not only in positive co-stimulatory pathways that increase lymphocyte activation, but also IRs that counterbalance these activation signals (20, 21).

While most strategies for countering T cell exhaustion focus on IRs restraining effector functions of CD8^+^ T cells, activated T cells constitutively express or upregulate numerous activating co-stimulatory molecules that can fine tune CD8^+^ T cell activation and are important for regulating CD8^+^ T cell responses to persisting infections and cancer. These receptors present potential therapeutic targets. However, analyses of these pathways also highlight the complex nature of co-stimulation in the development and function of Tex cells, where promoting activation can invigorate cells but may also paradoxically promote exhaustion.

### The Importance of Being There: CD226 in T Cell Exhaustion

CD226 (DNAX accessory molecule 1, DNAM-1) is expressed on T cells and contributes to cytotoxic lymphocyte (CTL) activation (22, 23). Studies examining CD226-deficient mice indicated that CD226 serves as a co-stimulatory receptor that amplifies CTL and NK cell-mediated cytotoxicity against targets expressing its ligands CD112 and CD155 (24, 25).

In chronic infection with HIV-1 and HIV-2, downregulation of CD226 has been observed in the peripheral blood: increased CD226^-^PD-1^+^ antigen-specific CD8^+^ T cells correlated with increased viral load (26, 27). CD226 downregulation also occurs in antigen-specific CD8^+^ T cells in mice chronically infected with LCMV clone 13, a common model used to evaluate T cell exhaustion. These studies suggest that Tex cells lose CD226 expression; however, the functional consequences remained unclear.

Through complementary experiments involving human samples and mouse tumor models, two groups showed that loss of CD226 induces hypo-responsiveness in CD8^+^ T cells, and limits both TCR signaling and responsiveness to anti-PD-1 mAbs (28, 29). CD226^-^CD8^+^ T cells failed to proliferate and produce effector cytokines upon CD3/CD28 mAbs stimulation, whereas ectopic re-expression of CD226 in CD226^-^CD8^+^ cells rescued responsiveness. Single-cell RNA sequencing (RNAseq) of tumor-specific CD8^+^ T cells isolated from murine melanomas revealed that CD226^+^ Tumor Infiltrating Lymphocytes (TILs) exhibited an enrichment for genes associated with T cell activation and immune synapse formation compared to CD226^-^ counterparts (28). Of note, decreased TCR signaling is observed in CD8^+^ Tex cells during chronic infection, as evidenced by low expression of the *Nr4a1*-GFP reporter and RNAseq analysis of TCR signaling-associated genes (30).

However, RNAseq comparing CD226^+^ and CD226^-^ CD8^+^ T cells post CD3/CD28 mAbs stimulation revealed that activated CD226^-^CD8^+^ T cells exhibited increased expression of genes associated with pTex cells including *Tcf7*, *Slamf6* (encoding TCF-1 and Ly108, respectively), as well as increased *Tox*. Nonetheless, one principal characteristic of pTex cells, the ability to response to ICB, is not shared by CD226^-^ cells. Anti-PD-1 mAbs failed to restore effector functions of TILs lacking CD226 expression in a transplantable melanoma model, suggesting that this Tex population resembles a more terminally exhausted population: a recent study confirmed that CD226 expression is required on TILs for efficient ICB responsiveness (31). Indeed, a continuum of low, intermediate, and high CD226 expression within PD-1^+^CD39^+^CD8^+^ exhausted TILs has been described. High CD226 expression correlated with increased ability of CD8^+^ TILs to secrete IFN-γ, suggesting that CD226 expression defines functional states among Tex and potentially more cytotoxic cells (28). These studies provide support that the absence of CD226 represents an unappreciated mechanism limiting TIL responsiveness independently of IR expression.

How is CD226 expression regulated? Post-translationally, the CD226 ligand, CD155, within the tumor microenvironment has been implicated in the downregulation of CD226 on TILs. Furthermore, mice with a Y319F mutation in CD226, which abrogates recruitment of the E3 ubiquitin ligase CBL-B for ubiquitinylation and proteasomal degradation, exhibited increased CD226 expression on CD8^+^ T cells and suppressed tumor growth (28). Additionally, CD226 downregulation was found to be dependent on the transcription factor Eomesodermin (Eomes), which is associated with T cell exhaustion (**Figure 1**) (32–34); increased Eomes was observed in CD226^-^CD8^+^ cells compared to CD226^+^ counterparts upon *in vitro* activation. In contrast, the absence of CD226 on CD4^+^ T cells is associated with decreased T-bet, a closely related transcription factor associated with IFN-γ and effector function; similarly, in NK cells, CD226 induces T-bet (35). Whether CD226 directly affects these transcription factors in CD8^+^ T cells is unclear. It is therefore of interest that another co-activating receptor, CD137 (4-1BB, TNFRSF9), has been implicated in the induction of Eomes and downmodulation of CD226 (29).

### CD137 – Too Much of a Good Thing

CD137 was initially described as a co-stimulatory member of the tumor necrosis factor receptor (TNFR) superfamily that enhanced T cell proliferation and cytokine secretion, as well as protected T cells from activation–induced cell death (36–38). Injection of CD137 agonist mAbs expanded CD8^+^ effector T memory cells and promoted tumor regression in a variety of mouse tumor models in a CD8^+^ T cell-dependent manner (39, 40), suggesting CD137 is a promising target to increase T cell function. In mouse tumor models, CD137 stimulation increased cytotoxicity of CD8^+^ TILs through increased Eomes expression (41). However, anti-CD137 mAb stimulation also promoted accumulation of dysfunctional CD226^-^CD8^+^ T cells in C57BL/6 WT mice (**Figure 1**).

Similar to hypofunctional CD226^-^CD8^+^ T cells within tumors, CD226^-^CD8^+^ T cells induced by anti-CD137 mAbs in C57BL/6 WT mice failed to proliferate and secrete TNF-α and IFN-γ in response to TCR stimulation. CD226^-^CD8^+^ TCR transgenic T cells generated in response to CD137 stimulation were devoid of effector functions after antigen stimulation *in vitro* and had significantly weaker anti-tumor properties than CD226^-^CD8^+^ T cells *in vivo*. Thus, hypofunctional CD226^-^CD8^+^ T cells are generated both in a tumor context and following CD137 stimulation in mice. In view of the expression of CD137 ligand (CD137L) by dendritic cells (42) and by numerous tumor lines (43, 44), CD137 may down-modulate CD226 both in early phases of anti-tumor immune responses and in later phases at the tumor site, respectively.

While the functions of CD137 may seem contradictory, CD137 ligation, similar to chronic infection, increases T cell activation, which may promote T cell exhaustion. These observations may provide insight into why anti–CD137 agonists decrease clinical symptoms in mouse models of autoimmunity including collagen-induced arthritis (45), experimental autoimmune uveoretinitis (45), EAE (46) and systemic lupus erythematosus (47). In addition, anti-CD137 mAbs can also activate NK cells (48, 49), macrophages (50) and inhibit Treg cells (51, 52), further complicating interpretation of their actions. Thus, these studies highlight the paradoxical roles of CD137 signaling in T cell exhaustion and the need for caution in clinical trials.

## Pharmacological Approaches to Manipulate Exhaustion

Pharmacological interventions, particularly those increasing or boosting TCF-1^+^ populations present alternative approaches to countering exhaustion, which are not focused on directly activating or inhibiting co-stimulatory molecules. In this regard, recent data on Phosphoinositide 3 Kinase delta (PI3Kδ present intriguing possibilities.

### PI3Kδ and the Regulation of CD8^+^ T Cell Function

PI3Ks are lipid kinases that catalyze the addition of phosphate to the D3 position of phosphoinositides, most notably the ubiquitous membrane phospholipid PI(_4,5_)P_2_, to generate PI(_3,4,5_)P_3_ (PIP_3_). PI3Kδ is highly expressed in hematopoietic cells and participates in signaling downstream from the TCR, CD28, ICOS, as well as chemokine and cytokine receptors. PIP_3_, in turn, recruits proteins containing Pleckstrin homology and other PIP_3_-binding domains to the membrane where they can interact with other proteins and/or be phosphorylated. Among its effectors, AKT kinases are key components of PI3K-activated pathways, phosphorylating downstream targets including transcription factors such as FoxO1 and BACH2, the chromatin modifier Ezh2, and regulators of mTOR (53). AKT-mediated phosphorylation leads to nuclear exclusion and inactivation of FoxO1 and BACH2. These signaling cascades can be counterbalanced by PD-1-mediated SHP1 recruitment which limits PI3K activation (54) as well as lipid phosphatases such as SHIP and PTEN (53).

**Figure 2:**
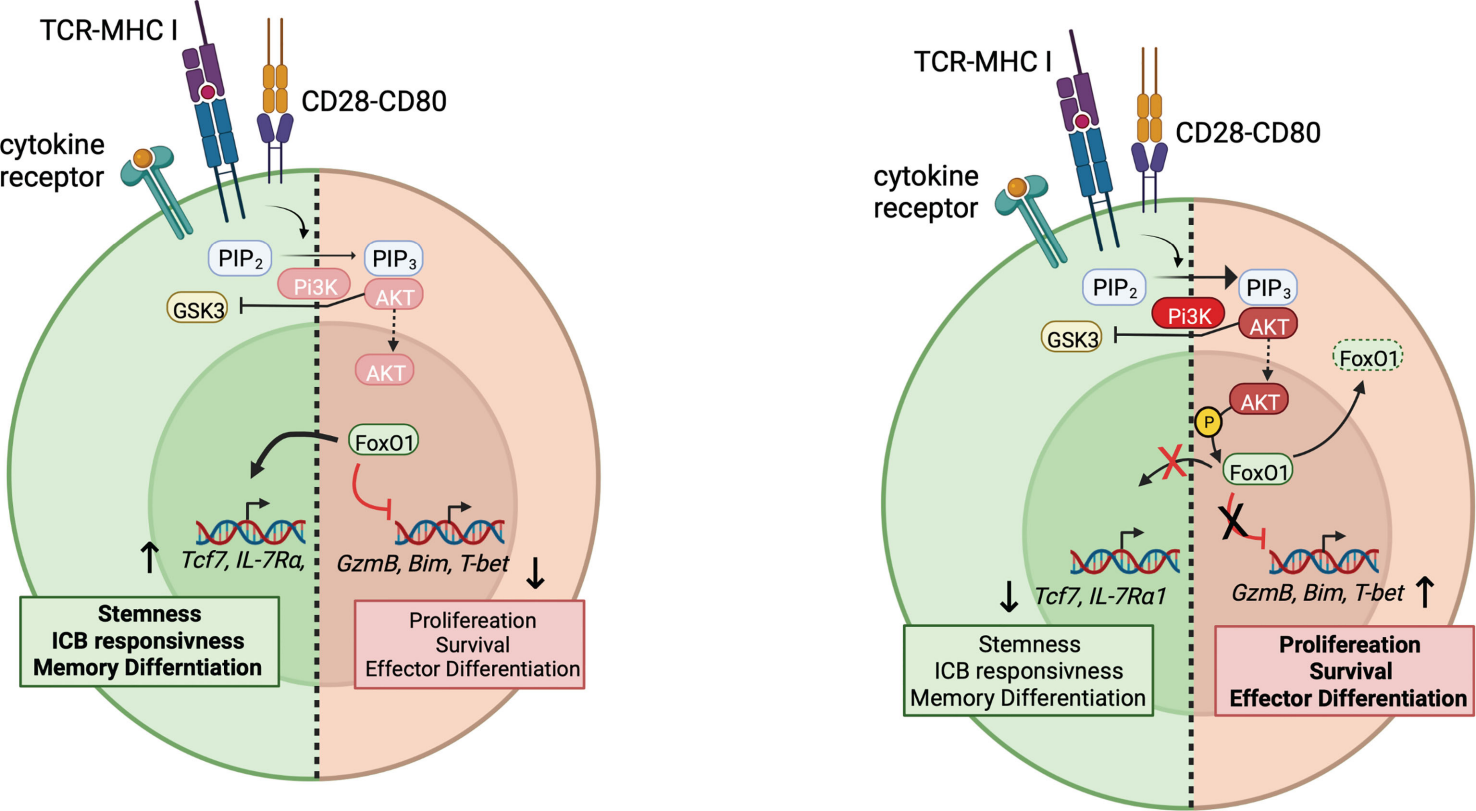
PI3K and regulation of stem-like pTex cells. Right: Upon T cell activation, PI3K generates PIP_3_, which recruits AKT, leading to its activation at the plasma membrane, which then leads to the phosphorylation and nuclear exclusion of FoxO1 and BACH2. Downstream genes associated with stemness and memory differentiation, such as *Tcf7* are no longer induced by FoxO1, whereas effector genes such as T-bet and GranzymeB, usually suppressed by FoxO1, can be transduced. How CD226 integrates in this model is not yet clear. Left: Under conditions of low PI3K activity, FoxO1 remains active, and *Tcf7* and other genes associated with pTex cells are expressed.

Recent data suggest that PI3K plays a major role in regulating expression of *Tcf7*, which is a FoxO1 transcriptional target (55). Evaluation of asymmetric T cell division *ex vivo*, revealed a bifurcation of TCF-1 expression: daughter cells that inherit robust PI3K activity inactivate FoxO1 and silence *Tcf7* expression. Daughter cells with reduced PI3K activity maintained TCF-1 and generated a self-renewing memory population (8, 56–58). Antagonism of PI3K activity *in vitro* limited repression of *Tcf7* and induction of differentiation markers (58). Conversely, recent work from our laboratory showed that expression of an activated PI3Kδ allele suppressed the maintenance of a TCF-1^+^CD8^+^ T cell population and the development of central memory cells during acute viral infection. Instead, activated PI3Kδ-expressing CD8^+^ T cells were driven to a long-lived effector cell fate with increased expression of effector cytokines IFN-γ and TNF-α (8). Similarly, T cells from patients with the immunodeficiency, Activated PI3K Delta Syndrome, failed to maintain a TCF-1^+^ population when expanded *in vitro* (8, 56). Together these studies raise the possibility that inhibition of PI3Kδ could promote expansion of TCF-1^+^ pTex, thereby increasing the population that can respond to ICB (**Figure 2**). Inhibition of PI3K would also be expected to prevent BACH2 inactivation, which also promotes pTex cells.

Nonetheless, PI3Kδ also plays an important role in both effector T and B cell differentiation (8, 59). Intriguingly, CD226-mediated induction of T-bet and cytotoxicity in NK cells occurs *via* FoxO1-mediated regulation (35); it is intriguing to speculate that CD226 engagement increases CD8^+^ T cell effector function *via* PI3K-mediated pathways. Thus, while PI3Kδ inhibition may increase TCF-1^+^ pTex, this may come at the expense of the ability to develop effector cells and even to respond to ICB (60). Manipulation of PI3Kδ pathways therefore raises multiple issues, including how to appropriately balance the promotion of TCF-1^+^ populations, while allowing development of effective cytolytic CD8^+^ T and other immune cell responses. Whether PI3Kδ inhbitors are useful during *ex vivo* expansion of TILs or CAR-T cells to maintain a TCF-1^+^ population that then can be transfered *in vivo* or further expanded without PI3K inhibition remains to be seen.

### IL-2: A Key Player in T Cell Differentiation and Function

Another approach that has been considered is therapeutic administration of cytokines such as IL-2, to reinvigorate Tex cells; however, this has yielded disparate results. *In vitro*, IL-2 drives the differentiation of cytolytic effector CD8^+^ T cells and the acquisition of effector functions (61, 62). Accordingly, following LCMV chronic infection, *in vivo* IL-2 treatment boosted the number of antigen specific Tex cells and improved viral control (60, 63). Interestingly, IL-2 treatment combined with PD-1 blockade had synergistic effects, perhaps through anti-PD-1 effects on pTex cells and IL-2 effects on promoting effector cells (60). However, IL-2 treatment can also result in the expansion of immunosuppressive Tregs, as well as vascular leakage syndrome and thus may have undesirable secondary effects (64, 65). IL-2-anti-IL2 complexes and stabilized forms of IL-2 also can have distinct effects on different cell-types.

While the effects of IL-2 and IL-2-complexes are beyond the scope of this review, recent data on IL-2 variants have yielded some intriguing results (63–66). An engineered pegylated-IL-2 variant, THOR-707, was found to selectively engage the IL-2R β/γ complex and have a longer half-life, leading to tumor reduction without Treg expansion (67). Another engineered IL-2 partial agonist, H9T, that also activates IL-2Rβ/γ, promoted CD8^+^ T cells with sustained TCF-1 expression and maintenance of a stem-like state, with higher spare respiratory capacity indicative of improved mitochondrial fitness (68). Although pAKT was unaffected under the conditions examined, it is interesting to speculate that H9T and other IL-2 variants may indirectly affect PI3K and thereby, FoxO1-mediated regulation of *Tcf7*.

These engineered IL-2-variants unveil promising strategies for boosting immunotherapeutic treatment regimes *via* promoting TCF-1^+^ pTex cells (65). Interestingly, data argue that following antigen stimulation, TCF-1 is required for the induction of glycolytic capacity of central memory T cells, which rely on fatty acid oxidation during memory phases in acute infection (69–71). TCF-1 expression in Tex cells may therefore be critical to maintain a state that can meet the continued bioenergetic demands in response sustained antigen exposure. Indeed, metabolic profiling revealed the importance of metabolism in Tex cell fate. Tex cells display metabolic insufficiency, including diminished glucose uptake and OXPHOS (72–74), resulting at least in-part from decreased expression of the transcriptional coactivator peroxisome proliferator-activated receptor gamma co-activator 1-alpha, PGC1α, which has critical roles in mitochondrial biogenesis and anti-oxidant responses (74). An *in vivo* CRISPR–Cas9 mutagenesis screen found that targeting the ribonuclease REGNASE-1 reprogrammed CD8^+^ T cells into long-lived effector cells with improved mitochondrial fitness and anti-tumor responses. These findings highlight the importance of mitochondrial quality, and potentially mitochondrial activity, in orchestrating T-cell function and fate during exhaustion (72, 74).

Nonetheless, Tex cells originating from a TCF-1^+^ population during chronic infection following antigen elimination (‘recovered’ cells) likely still remain compromised: these cells maintain features of an exhausted chromatin landscape and have been referred to as ‘epigentically scarred’ (15, 16, 75). Whether this contributes to the failure of checkpoint blockade in many cases is unknown. Thus, increasing the TCF-1^+^ population may not be sufficient as a therapeutic strategy; approaches to increase epigenetic plasticity of TCF-1^+^CD8^+^ T cells remain an important area for further investigation. Such approaches could involve dampening chronic inflammation (16) as well as preventing nutrient deprivation and/or elimination deleterious metabolities (73, 76, 77).

## Not All Roads Lead to Rome – Routes to Prevent Exhaustion

In a striking report, intravenous vaccination using a TLR7/9 agonist in conjunction with nanopartical presentation of a neoantigen induced a higher proportion of stem-like CD8^+^ T cells compared to subcutaneous immunization. Moreover, the stem-like TCF-1^+^ cells generated were able to differentiate into effector CD8^+^ T cells to elicit anti-tumour responses (78). Similarly, the duration of antigen presentation by specific dendritic populations in the draining lymph node and spleen may help augment and/or maintain the reservoir of TCF-1^+^CD8^+^ T cells required for optimal immunity during chronic antigen exposure (79–81). Such reports suggest that routes and types of immunizations can elicit responses that facilitate the development of successful tumor vaccines to overcome limitations of exhaustion. Whether these approaches can work in combination for chronic infection and cancer, whether they affect some of the pathways described above, including activation of PI3K, IL-2 or costimulatory pathways and what other techniques may be used to boost the long-lived potential of adoptive cell therapies remain important questions.

## Summary

The study of T cell exhaustion and the development of ICB has moved rapidly in recent years. However, while the success of immune-based therapies has been striking, the successful implementation in only a fraction of cancers, argues that new approaches are needed. Here, we reviewed several novel approaches including stimulation of activating receptors and pharmacological methods to increase TCF-1^+^ pTex cells. Nonetheless, these data highlight the complexity of T cell exhaustion and the fragile balance between T cell activation and exhaustion. Combinations that recognize these limitations while taking advantage of distinct features of these approaches may ultimately help improve the success of immunotherapy.

## Author Contributions

ACP, JLC and PLS wrote and edited the manuscript. ACP designed and generated the figures with feedback from JLC and PLS. All authors contributed to the article and approved the submitted version.

## Funding

This work was supported by the Intramural Research Program of NIAID, NIH.

## Conflict of Interest

The authors declare that the research was conducted in the absence of any commercial or financial relationships that could be construed as a potential conflict of interest.

## Publisher’s Note

All claims expressed in this article are solely those of the authors and do not necessarily represent those of their affiliated organizations, or those of the publisher, the editors and the reviewers. Any product that may be evaluated in this article, or claim that may be made by its manufacturer, is not guaranteed or endorsed by the publisher.

## References

[1] Jameson SC Masopust D. Understanding Subset Diversity in T Cell Memory. Immunity (2018) 48:214–26. doi: 10.1016/j.immuni.2018.02.010 PMC5863745 29466754

[2] Topalian SL Hodi FS Brahmer JR Gettinger SN Smith DC McDermott DF. Safety, Activity, and Immune Correlates of Anti–PD-1 Antibody in Cancer. N Engl J Med (2012) 366:2443–54. doi: 10.1056/NEJMoa1200690 PMC3544539 22658127

[3] Robert C Schachter J Long GV Arance A Grob JJ Mortier L. Pembrolizumab Versus Ipilimumab in Advanced Melanoma. N Engl J Med (2015) 372:2521–32. doi: 10.1056/NEJMoa1503093 25891173

[4] McLane LM Abdel-Hakeem MS Wherry EJ. CD8 T Cell Exhaustion During Chronic Viral Infection and Cancer. Annu Rev Immunol (2019) 37:457–95. doi: 10.1146/annurev-immunol-041015-055318 30676822

[5] Chen Z Ji Z Ngiow SF Manne S Cai Z Huang AC. TCF-1-Centered Transcriptional Network Drives an Effector Versus Exhausted CD8 T Cell-Fate Decision. Immunity (2019) 51:840–55.e5. doi: 10.1016/j.immuni.2019.09.013 31606264 PMC6943829

[6] Wu T Ji Y Moseman EA Xu HC Manglani M Kirby M. The TCF1-Bcl6 Axis Counteracts Type I Interferon to Repress Exhaustion and Maintain T Cell Stemness. Sci Immunol (2016) 1:eaai8593–eaai8593. doi: 10.1126/sciimmunol.aai8593 28018990 PMC5179228

[7] Wang Y Hu J Li Y Xiao M Wang H Tian Q. The Transcription Factor TCF1 Preserves the Effector Function of Exhausted CD8 T Cells During Chronic Viral Infection. Front Immunol (2019) 10:169. doi: 10.3389/fimmu.2019.00169 30814995 PMC6381939

[8] Cannons JL Villarino AV Kapnick SM Preite S Shih H-Y Gomez-Rodriguez J. Pi3kδ Coordinates Transcriptional, Chromatin, and Metabolic Changes to Promote Effector CD8+ T Cells at the Expense of Central Memory. Cell Rep (2021) 37:109804. doi: 10.1016/j.celrep.2021.109804 34644563 PMC8582080

[9] Zander R Schauder D Xin G Nguyen C Wu X Zajac A. CD4+ T Cell Help Is Required for the Formation of a Cytolytic CD8+ T Cell Subset That Protects Against Chronic Infection and Cancer. Immunity (2019) 51:1028–42.e4. doi: 10.1016/j.immuni.2019.10.009 31810883 PMC6929322

[10] Beltra J-C Manne S Abdel-Hakeem MS Kurachi M Giles JR Chen Z. Developmental Relationships of Four Exhausted CD8+ T Cell Subsets Reveals Underlying Transcriptional and Epigenetic Landscape Control Mechanisms. Immunity (2020) 52:825–41.e8. doi: 10.1016/j.immuni.2020.04.014 32396847 PMC8360766

[11] Im SJ Hashimoto M Gerner MY Lee J Kissick HT Burger MC. Defining CD8+ T Cells That Provide the Proliferative Burst After PD-1 Therapy. Nature (2016) 537:417–21. doi: 10.1038/nature19330 PMC5297183 27501248

[12] Khan O Giles JR McDonald S Manne S Ngiow SF Patel KP. TOX Transcriptionally and Epigenetically Programs CD8+ T Cell Exhaustion. Nature (2019) 571:211–8. doi: 10.1038/s41586-019-1325-x PMC6713202 31207603

[13] Scott AC Dündar F Zumbo P Chandran SS Klebanoff CA Shakiba M. TOX Is a Critical Regulator of Tumour-Specific T Cell Differentiation. Nature (2019) 571:270–4. doi: 10.1038/s41586-019-1324-y PMC7698992 31207604

[14] Alfei F Kanev K Hofmann M Wu M Ghoneim HE Roelli P. TOX Reinforces the Phenotype and Longevity of Exhausted T Cells in Chronic Viral Infection. Nature (2019) 571:265–9. doi: 10.1038/s41586-019-1326-9 31207605

[15] Abdel-Hakeem MS Manne S Beltra J-C Stelekati E Chen Z Nzingha K. Epigenetic Scarring of Exhausted T Cells Hinders Memory Differentiation Upon Eliminating Chronic Antigenic Stimulation. Nat Immunol (2021) 22:1008–19. doi: 10.1038/s41590-021-00975-5 PMC8323971 34312545

[16] Yates KB Tonnerre P Martin GE Gerdemann U Al Abosy R Comstock DE. Epigenetic Scars of CD8+ T Cell Exhaustion Persist After Cure of Chronic Infection in Humans. Nat Immunol (2021) 22:1020–9. doi: 10.1038/s41590-021-00979-1 PMC8600539 34312547

[17] Hensel N Gu Z Sagar Wieland D Jechow K Kemming J. Memory-Like HCV-Specific CD8+ T Cells Retain a Molecular Scar After Cure of Chronic HCV Infection. Nat Immunol (2021) 22:229–39. doi: 10.1038/s41590-020-00817-w 33398179

[18] Yao C Lou G Sun H-W Zhu Z Sun Y Chen Z. BACH2 Enforces the Transcriptional and Epigenetic Programs of Stem-Like CD8+ T Cells. Nat Immunol (2021) 22:370–80. doi: 10.1038/s41590-021-00868-7 PMC7906956 33574619

[19] Utzschneider DT Gabriel SS Chisanga D Gloury R Gubser PM Vasanthakumar A. Early Precursor T Cells Establish and Propagate T Cell Exhaustion in Chronic Infection. Nat Immunol (2020) 21:1256–66. doi: 10.1038/s41590-020-0760-z 32839610

[20] Greenwald RJ Freeman GJ Sharpe AH. THE B7 FAMILY REVISITED. Annu Rev Immunol (2005) 23:515–48. doi: 10.1146/annurev.immunol.23.021704.115611 15771580

[21] Odorizzi PM Wherry EJ. Inhibitory Receptors on Lymphocytes: Insights From Infections. J Immunol (2012) 188:2957–65. doi: 10.4049/jimmunol.1100038 PMC3320038 22442493

[22] Burns GF Triglia T Werkmeister JA Begley CG Boyd AW. TLiSA1, a Human T Lineage-Specific Activation Antigen Involved in the Differentiation of Cytotoxic T Lymphocytes and Anomalous Killer Cells From Their Precursors. J Exp Med (1985) 161:1063–78. doi: 10.1084/jem.161.5.1063 PMC2187589 2580933

[23] Scott JL Dunn SM Jin B Hillam AJ Walton S Berndt MC. Characterization of a Novel Membrane Glycoprotein Involved in Platelet Activation. J Biol Chem (1989) 264:13475–82. doi: 10.1016/S0021-9258(18)80021-7 2760031

[24] Gilfillan S Chan CJ Cella M Haynes NM Rapaport AS Boles KS. DNAM-1 Promotes Activation of Cytotoxic Lymphocytes by Nonprofessional Antigen-Presenting Cells and Tumors. J Exp Med (2008) 205:2965–73. doi: 10.1084/jem.20081752 PMC2605240 19029380

[25] Martinet L Smyth MJ. Balancing Natural Killer Cell Activation Through Paired Receptors. Nat Rev Immunol (2015) 15:243–54. doi: 10.1038/nri3799 25743219

[26] Cella M Presti R Vermi W Lavender K Turnbull E Ochsenbauer-Jambor C. Loss of DNAM-1 Contributes to CD8 ^+^ T-Cell Exhaustion in Chronic HIV-1 Infection: HIGHLIGHTS. Eur J Immunol (2010) 40:949–54. doi: 10.1002/eji.200940234 PMC3031090 20201043

[27] Scharf L Pedersen CB Johansson E Lindman J Olsen LR Buggert M. Inverted CD8 T-Cell Exhaustion and Co-Stimulation Marker Balance Differentiate Aviremic HIV-2-Infected From Seronegative Individuals. Front Immunol (2021) 12:744530. doi: 10.3389/fimmu.2021.744530 34712231 PMC8545800

[28] Braun M Aguilera AR Sundarrajan A Corvino D Stannard K Krumeich S. CD155 on Tumor Cells Drives Resistance to Immunotherapy by Inducing the Degradation of the Activating Receptor CD226 in CD8+ T Cells. Immunity (2020) 53:805–23.e15. doi: 10.1016/j.immuni.2020.09.010 33053330

[29] Weulersse M Asrir A Pichler AC Lemaitre L Braun M Carrié N. Eomes-Dependent Loss of the Co-Activating Receptor CD226 Restrains CD8+ T Cell Anti-Tumor Functions and Limits the Efficacy of Cancer Immunotherapy. Immunity (2020) 53:824–39.e10. doi: 10.1016/j.immuni.2020.09.006 33053331

[30] Sandu I Cerletti D Claassen M Oxenius A. Exhausted CD8+ T Cells Exhibit Low and Strongly Inhibited TCR Signaling During Chronic LCMV Infection. Nat Commun (2020) 11:4454. doi: 10.1038/s41467-020-18256-4 32901001 PMC7479152

[31] Banta KL Xu X Chitre AS Au-Yeung A Takahashi C O’Gorman WE. Mechanistic Convergence of the TIGIT and PD-1 Inhibitory Pathways Necessitates Co-Blockade to Optimize Anti-Tumor CD8+ T Cell Responses. Immunity (2022) 55:512–26.e9. doi: 10.1016/j.immuni.2022.02.005 35263569 PMC9287124

[32] Buggert M Tauriainen J Yamamoto T Frederiksen J Ivarsson MA Michaëlsson J. T-Bet and Eomes Are Differentially Linked to the Exhausted Phenotype of CD8+ T Cells in HIV Infection. PLos Pathog (2014) 10:e1004251. doi: 10.1371/journal.ppat.1004251 25032686 PMC4102564

[33] McLane LM Ngiow SF Chen Z Attanasio J Manne S Ruthel G. Role of Nuclear Localization in the Regulation and Function of T-Bet and Eomes in Exhausted CD8 T Cells. Cell Rep (2021) 35:109120. doi: 10.1016/j.celrep.2021.109120 33979613 PMC8195461

[34] Li J He Y Hao J Ni L Dong C. High Levels of Eomes Promote Exhaustion of Anti-Tumor CD8+ T Cells. Front Immunol (2018) 9:2981. doi: 10.3389/fimmu.2018.02981 30619337 PMC6305494

[35] Du X de Almeida P Manieri N de Almeida Nagata D Wu TD Harden Bowles K. CD226 Regulates Natural Killer Cell Antitumor Responses *via* Phosphorylation-Mediated Inactivation of Transcription Factor FOXO1. Proc Natl Acad Sci USA (2018) 115(50):E11731–40. doi: 10.1073/pnas.1814052115 PMC6294892 30504141

[36] Hurtado JC Kim YJ Kwon BS. Signals Through 4-1BB are Costimulatory to Previously Activated Splenic T Cells and Inhibit Activation-Induced Cell Death. J Immunol (1997) 158:2600–9. 9058792

[37] Shuford WW Klussman K Tritchler DD Loo DT Chalupny J Siadak AW. 4-1bb Costimulatory Signals Preferentially Induce CD8+ T Cell Proliferation and Lead to the Amplification *In Vivo* of Cytotoxic T Cell Responses. J Exp Med (1997) 186:47–55. doi: 10.1084/jem.186.1.47 9206996 PMC2198949

[38] Wilcox RA Chapoval AI Gorski KS Otsuji M Shin T Flies DB. Cutting Edge: Expression of Functional CD137 Receptor by Dendritic Cells. J Immunol (2002) 168:4262–7. doi: 10.4049/jimmunol.168.9.4262 11970964

[39] Guillerey C Ferrari de Andrade L Vuckovic S Miles K Ngiow SF Yong MCR. Immunosurveillance and Therapy of Multiple Myeloma Are CD226 Dependent. J Clin Invest. (2015) 125:2077–89. doi: 10.1172/JCI77181 PMC4463191 25893601

[40] Melero I Shuford WW Newby SA Aruffo A Ledbetter JA Hellström KE. Monoclonal Antibodies Against the 4-1BB T-Cell Activation Molecule Eradicate Established Tumors. Nat Med (1997) 3:682–5. doi: 10.1038/nm0697-682 9176498

[41] Curran MA Geiger TL Montalvo W Kim M Reiner SL Al-Shamkhani A. Systemic 4-1BB Activation Induces a Novel T Cell Phenotype Driven by High Expression of Eomesodermin. J Exp Med (2013) 210:743–55. doi: 10.1084/jem.20121190 PMC3620352 23547098

[42] Shao Z Schwarz H. CD137 Ligand, a Member of the Tumor Necrosis Factor Family, Regulates Immune Responses *via* Reverse Signal Transduction. J Leukocyte Biol (2011) 89:21–9. doi: 10.1189/jlb.0510315 20643812

[43] Salih HR Kosowski SG Haluska VF Starling GC Loo DT Lee F. Constitutive Expression of Functional 4-1bb (CD137) Ligand on Carcinoma Cells. J Immunol (2000) 165:2903–10. doi: 10.4049/jimmunol.165.5.2903 10946324

[44] Wang Q Zhang P Zhang Q Wang X Li J Ma C. Analysis of CD137 and CD137L Expression in Human Primary Tumor Tissues. Croat Med J (2008) 49:192–200. doi: 10.3325/cmj.2008.2.192 18461674 PMC2359873

[45] Seo SK Choi JH Kim YH Kang WJ Park HY Suh JH. 4-1BB-Mediated Immunotherapy of Rheumatoid Arthritis. Nat Med (2004) 10:1088–94. doi: 10.1038/nm1107 15448685

[46] Sun Y Lin X Chen HM Wu Q Subudhi SK Chen L. Administration of Agonistic Anti-4-1bb Monoclonal Antibody Leads to the Amelioration of Experimental Autoimmune Encephalomyelitis. J Immunol (2002) 168:1457–65. doi: 10.4049/jimmunol.168.3.1457 11801689

[47] Foell J Strahotin S O’Neil SP McCausland MM Suwyn C Haber M. CD137 Costimulatory T Cell Receptor Engagement Reverses Acute Disease in Lupus-Prone NZB × NZW F1 Mice. J Clin Invest. (2003) 111:1505–18. doi: 10.1172/JCI200317662 PMC155050 12750400

[48] Wilcox RA Tamada K Strome SE Chen L. Signaling Through NK Cell-Associated CD137 Promotes Both Helper Function for CD8 ^+^ Cytolytic T Cells and Responsiveness to IL-2 But Not Cytolytic Activity. J Immunol (2002) 169:4230–6. doi: 10.4049/jimmunol.169.8.4230 12370353

[49] Misumi T Tanabe K Fujikuni N Ohdan H. Stimulation of Natural Killer Cells With Rhcd137 Ligand Enhances Tumor-Targeting Antibody Efficacy in Gastric Cancer. PLos One (2018) 13:e0204880. doi: 10.1371/journal.pone.0204880 30321186 PMC6188629

[50] Stoll A Bruns H Fuchs M Völkl S Nimmerjahn F Kunz M. CD137 (4-1BB) Stimulation Leads to Metabolic and Functional Reprogramming of Human Monocytes/Macrophages Enhancing Their Tumoricidal Activity. Leukemia (2021) 35:3482–96. doi: 10.1038/s41375-021-01287-1 PMC8632678 34021248

[51] Akhmetzyanova I Zelinskyy G Littwitz-Salomon E Malyshkina A Dietze KK Streeck H. CD137 Agonist Therapy Can Reprogram Regulatory T Cells Into Cytotoxic CD4 ^+^ T Cells With Antitumor Activity. J Immunol (2016) 196:484–92. doi: 10.4049/jimmunol.1403039 26608920

[52] Smith SE Hoelzinger DB Dominguez AL Van Snick J Lustgarten J. Signals Through 4-1BB Inhibit T Regulatory Cells by Blocking IL-9 Production Enhancing Antitumor Responses. Cancer Immunol Immunother (2011) 60:1775–87. doi: 10.1007/s00262-011-1075-6 PMC3919703 21789593

[53] Okkenhaug K Vanhaesebroeck B. PI3K in Lymphocyte Development, Differentiation and Activation. Nat Rev Immunol (2003) 3:317–30. doi: 10.1038/nri1056 12669022

[54] Sharpe AH. Mechanisms of Costimulation. Immunol Rev (2009) 229:5–11. doi: 10.1111/j.1600-065X.2009.00784.x 19426211 PMC2928676

[55] Delpoux A Lai C-Y Hedrick SM Doedens AL. FOXO1 Opposition of CD8 ^+^ T Cell Effector Programming Confers Early Memory Properties and Phenotypic Diversity. Proc Natl Acad Sci USA (2017) 114(42):E8865–74. doi: 10.1073/pnas.1618916114 PMC5651728 28973925

[56] Chen Y-H Kratchmarov R Lin W-HW Rothman NJ Yen B Adams WC. Asymmetric PI3K Activity in Lymphocytes Organized by a PI3K-Mediated Polarity Pathway. Cell Rep (2018) 22:860–8. doi: 10.1016/j.celrep.2017.12.087 PMC5806629 29420173

[57] Lin W-HW Nish SA Yen B Chen Y-H Adams WC Kratchmarov R. CD8 + T Lymphocyte Self-Renewal During Effector Cell Determination. Cell Rep (2016) 17:1773–82. doi: 10.1016/j.celrep.2016.10.032 PMC5108530 27829149

[58] Lin W-HW Adams WC Nish SA Chen Y-H Yen B Rothman NJ. Asymmetric PI3K Signaling Driving Developmental and Regenerative Cell Fate Bifurcation. Cell Rep (2015) 13:2203–18. doi: 10.1016/j.celrep.2015.10.072 PMC4685001 26628372

[59] Lim EL Cugliandolo FM Rosner DR Gyori D Roychoudhuri R Okkenhaug K. Phosphoinositide 3-Kinase δ Inhibition Promotes Antitumor Responses But Antagonizes Checkpoint Inhibitors. JCI Insight (2018) 3:e120626. doi: 10.1172/jci.insight.120626 PMC6124416 29875319

[60] West EE Jin H-T Rasheed A-U Penaloza-MacMaster P Ha S-J Tan WG. PD-L1 Blockade Synergizes With IL-2 Therapy in Reinvigorating Exhausted T Cells. J Clin Invest (2013) 123:2604–15. doi: 10.1172/JCI67008 PMC3668811 23676462

[61] Pipkin ME Sacks JA Cruz-Guilloty F Lichtenheld MG Bevan MJ Rao A. Interleukin-2 and Inflammation Induce Distinct Transcriptional Programs That Promote the Differentiation of Effector Cytolytic T Cells. Immunity (2010) 32:79–90. doi: 10.1016/j.immuni.2009.11.012 20096607 PMC2906224

[62] Kalia V Sarkar S. Regulation of Effector and Memory CD8 T Cell Differentiation by IL-2—A Balancing Act. Front Immunol (2018) 9:2987. doi: 10.3389/fimmu.2018.02987 30619342 PMC6306427

[63] Blattman JN Grayson JM Wherry EJ Kaech SM Smith KA Ahmed R. Therapeutic Use of IL-2 to Enhance Antiviral T-Cell Responses *In Vivo* . Nat Med (2003) 9:540–7. doi: 10.1038/nm866 12692546

[64] Boyman O Surh CD Sprent J. Potential Use of IL-2/Anti-IL-2 Antibody Immune Complexes for the Treatment of Cancer and Autoimmune Disease. Expert Opin Biol Ther (2006) 6:1323–31. doi: 10.1517/14712598.6.12.1323 17223740

[65] Hernandez R Põder J La Porte KM Malek TR. Engineering IL-2 for Immunotherapy of Autoimmunity and Cancer. Nat Rev Immunol (2022) 286(3):897–908. doi: 10.1038/s41577-022-00680-w 35217787

[66] Sun Z Ren Z Yang K Liu Z Cao S Deng S. A Next-Generation Tumor-Targeting IL-2 Preferentially Promotes Tumor-Infiltrating CD8+ T-Cell Response and Effective Tumor Control. Nat Commun (2019) 10:3874. doi: 10.1038/s41467-019-11782-w 31462678 PMC6713724

[67] Ptacin JL Caffaro CE Ma L San Jose Gall KM Aerni HR Acuff NV. An Engineered IL-2 Reprogrammed for Anti-Tumor Therapy Using a Semi-Synthetic Organism. Nat Commun (2021) 12:4785. doi: 10.1038/s41467-021-24987-9 34373459 PMC8352909

[68] Mo F Yu Z Li P Oh J Spolski R Zhao L. An Engineered IL-2 Partial Agonist Promotes CD8+ T Cell Stemness. Nature (2021) 597:544–8. doi: 10.1038/s41586-021-03861-0 PMC9172917 34526724

[69] Shan Q Hu SS Zhu S Chen X Badovinac VP Peng W. Tcf1 Preprograms the Mobilization of Glycolysis in Central Memory CD8+ T Cells During Recall Responses. Nat Immunol (2022) 23:386–98. doi: 10.1038/s41590-022-01131-3 PMC8904300 35190717

[70] Zhou X Yu S Zhao D-M Harty JT Badovinac VP Xue H-H. Differentiation and Persistence of Memory CD8+ T Cells Depend on T Cell Factor 1. Immunity (2010) 33:229–40. doi: 10.1016/j.immuni.2010.08.002 PMC2928475 20727791

[71] Jeannet G Boudousquié C Gardiol N Kang J Huelsken J Held W. Essential Role of the Wnt Pathway Effector Tcf-1 for the Establishment of Functional CD8 T Cell Memory. Proc Natl Acad Sci USA (2010) 107:9777–82. doi: 10.1073/pnas.0914127107 PMC2906901 20457902

[72] Bengsch B Johnson AL Kurachi M Odorizzi PM Pauken KE Attanasio J. Bioenergetic Insufficiencies Due to Metabolic Alterations Regulated by the Inhibitory Receptor PD-1 Are an Early Driver of CD8 + T Cell Exhaustion. Immunity (2016) 45:358–73. doi: 10.1016/j.immuni.2016.07.008 PMC4988919 27496729

[73] Schurich A Pallett LJ Jajbhay D Wijngaarden J Otano I Gill US. Distinct Metabolic Requirements of Exhausted and Functional Virus-Specific CD8 T Cells in the Same Host. Cell Rep (2016) 16:1243–52. doi: 10.1016/j.celrep.2016.06.078 PMC4977274 27452473

[74] Scharping NE Menk AV Moreci RS Whetstone RD Dadey RE Watkins SC. The Tumor Microenvironment Represses T Cell Mitochondrial Biogenesis to Drive Intratumoral T Cell Metabolic Insufficiency and Dysfunction. Immunity (2016) 45:374–88. doi: 10.1016/j.immuni.2016.07.009 PMC5207350 27496732

[75] Tonnerre P Wolski D Subudhi S Aljabban J Hoogeveen RC Damasio M. Differentiation of Exhausted CD8+ T Cells After Termination of Chronic Antigen Stimulation Stops Short of Achieving Functional T Cell Memory. Nat Immunol (2021) 22:1030–41. doi: 10.1038/s41590-021-00982-6 PMC8323980 34312544

[76] Fisicaro P Barili V Montanini B Acerbi G Ferracin M Guerrieri F. Targeting Mitochondrial Dysfunction can Restore Antiviral Activity of Exhausted HBV-Specific CD8 T Cells in Chronic Hepatitis B. Nat Med (2017) 23:327–36. doi: 10.1038/nm.4275 28165481

[77] Vodnala SK Eil R Kishton RJ Sukumar M Yamamoto TN Ha N-H. T Cell Stemness and Dysfunction in Tumors are Triggered by a Common Mechanism. Science (2019) 363:eaau0135. doi: 10.1126/science.aau0135 30923193 PMC8194369

[78] Baharom F Ramirez-Valdez RA Tobin KKS Yamane H Dutertre C-A Khalilnezhad A. Intravenous Nanoparticle Vaccination Generates Stem-Like TCF1+ Neoantigen-Specific CD8+ T Cells. Nat Immunol (2021) 22:41–52. doi: 10.1038/s41590-020-00810-3 33139915 PMC7746638

[79] Schenkel JM Herbst RH Canner D Li A Hillman M Shanahan S-L. Conventional Type I Dendritic Cells Maintain a Reservoir of Proliferative Tumor-Antigen Specific TCF-1+ CD8+ T Cells in Tumor-Draining Lymph Nodes. Immunity (2021) 54:2338–53.e6. doi: 10.1016/j.immuni.2021.08.026 34534439 PMC8604155

[80] Marangoni F Zhakyp A Corsini M Geels SN Carrizosa E Thelen M. Expansion of Tumor-Associated Treg Cells Upon Disruption of a CTLA-4-Dependent Feedback Loop. Cell (2021) 184:3998–4015.e19. doi: 10.1016/j.cell.2021.05.027 34157302 PMC8664158

[81] Dähling S Mansilla AM Knöpper K Grafen A Utzschneider DT Ugur M. Type 1 Conventional Dendritic Cells Maintain and Guide the Differentiation of Precursors of Exhausted T Cells in Distinct Cellular Niches. Immunity (2022) 55:656–70.e8. doi: 10.1016/j.immuni.2022.03.006 35366396

